# Incidental Ovarian Vein Thrombosis During Breast Cancer Therapy With Tamoxifen and Trastuzumab

**DOI:** 10.7759/cureus.92955

**Published:** 2025-09-22

**Authors:** Jorge Illarramendi, Susana Mauleon, Helena Sarasibar, Jose Juan Illarramendi

**Affiliations:** 1 Hematology, Hôpital Haut-Lévêque, Pessac, FRA; 2 Radiology, Hospital Universitario de Navarra, Pamplona, ESP; 3 Medical Oncology, Hospital Universitario de Navarra, Pamplona, ESP

**Keywords:** anticoagulation, breast cancer, ovarian vein thrombosis, tamoxifen, trastuzumab

## Abstract

Ovarian vein thrombosis (OVT) is an unusual presentation of venous thromboembolic disease. Incidental asymptomatic appearance of OVT is a recognized complication of gynecologic cancer surgery, but published information on incidental OVT during targeted therapy of cancer with hormone therapy and/or monoclonal antibodies is scarce. We present the case of a 41-year-old woman who had a diagnosis of asymptomatic right OVT during the adjuvant treatment of breast cancer with tamoxifen plus trastuzumab. Clinical evolution in this case was favorable without anticoagulant therapy, with confirmed thrombus resolution and without further events. Available literature on this topic is limited, but some data indicate that anticoagulation is also a valuable option to consider, according to potential advantages and risks, on an individual basis.

## Introduction

Ovarian vein thrombosis (OVT) is a rare presentation of venous thromboembolic disease (VTD), with distinctive radiological findings and a growing incidence due to the increased use of imaging studies [1]. OVT is considered to be around 60 times less common than lower limb deep vein thrombosis, and is usually related to pregnancy, gynecologic surgery, pelvic infections, and malignancy [2]. Pathophysiology of OVT includes venous stasis, hypercoagulability, and endothelial injury. Direct spread of the thrombus may also involve the inferior vena cava and left renal vein.

There are scales for VTD risk calculation in cancer patients that include the antiestrogen tamoxifen (TMX) as a fairly recognized prothrombotic drug [3], but this is not the case for the monoclonal antibody trastuzumab (TZB), better known for its cardiotoxicity risks.

Herein, we describe a case of OVT that presented during the adjuvant treatment of breast cancer (BC) with TMX plus TZB.

## Case presentation

A 41-year-old woman, born and resident in Venezuela, was diagnosed with left BC. She migrated to Peru for treatment, where a segmentectomy plus axillary lymphadenectomy was performed in February 2021. Histopathology revealed a grade 2 infiltrating ductal carcinoma, phenotype luminal-Her2, with positive estrogen and progesterone receptors and overexpression of human epidermal receptor 2 (Her2/neu) in tumor cells. Pathologic stage was pT2N1aM0. She completed adjuvant chemotherapy with four cycles of docetaxel and cyclophosphamide at the end of May 2021. Additional treatments with the monoclonal antibody TZB, hormone therapy with TMX, and radiation therapy (RT) were recommended, but they were unaffordable for the patient. After a further migration to Spain, TZB and TMX were started at our center in October 2021. Exploration and breast imaging at that time disclosed a small suspicious nodule under the scar. Cytological aspiration revealed a local relapse of BC, with the same histopathological features as the previous diagnosis. Baseline blood tests before the start of therapy were normal except for iron deficiency anemia. Hemoglobin was 9 g/dl (reference range:12-16 g/dl), serum iron: 18 µg/dl (reference range: 50-170 µg/dl), ferritin: 6 µg/l (reference range: 10-204 µg/l). Supplemental iron was started, with a fast response. A transvaginal ultrasound study performed at the start of TMX was reported as normal for the uterus and ovaries.

**Figure 1:**
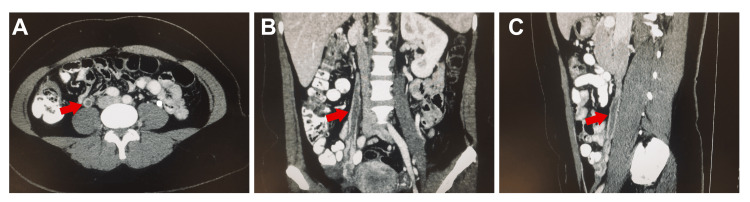
A) Axial, B) Coronal, C) Sagittal enhanced-CT views of right ovarian thrombosis (red arrows) A low-attenuation filling defect is present within the dilated vein.

Restaging with contrast-enhanced computed tomography (CT) scan was performed in November 2021, 47 days after the start of TZB and 43 days after the start of TMX. A BrightSpeed scanner (General Electric Medical Systems) was used, with a slice thickness of 1.25 millimeters. Contrast medium: Iodixanol. Abdominal image acquisition: 70 seconds after contrast infusion. There were no signs of metastases, but findings of right OVT were apparent in CT images (Figure 1).

**Figure 2:**
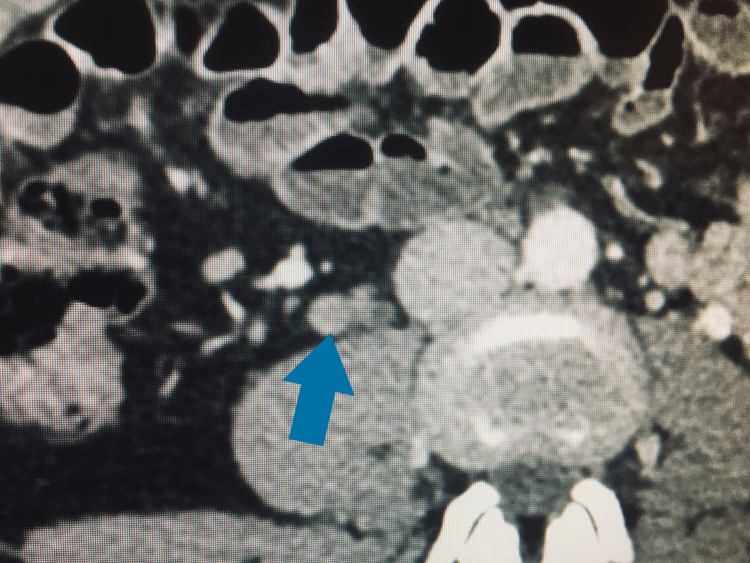
Follow-up CT scan showing an axial view of the ovarian vein (blue arrow) There is no filling defect. Vein diameter is smaller.

Diameter of right ovarian vein (axial view): 12 mm. Length of thrombus, including ovarian venous plexus: 124 millimeters. The patient was fully asymptomatic, without any abdominal or gynecologic complaints. Iron and ferritin levels were normal. Hemoglobin level was improved to 11 g/dl. D-dimer was 610 Fibrinogen Equivalent Units (FEU)/ml (reference range: 0-500 FEU/ml). A thrombophilia work-up, including homocysteine, antithrombin, lupus anticoagulant (Russell and Silica), factor V Leiden, and prothrombin gene mutations, yielded negative results. VTD risks were evaluated with specific assessment models for oncology patients, as follows: Khorana score 1 point (intermediate risk), COMPASS-CAT score 6 points (low-intermediate risk). She had no previous history of VTD. No symptoms related to thrombosis were present since the start of TMX and TZB. Anticoagulation was not prescribed, according to our institutional policy and following the guideline on incidental VTD from the International Society on Thrombosis and Hemostasis (ISTH), lack of known thrombophilia, and risk scores. The patient continued on TMX and TZB after surgical removal of the local relapse and postoperative irradiation. No signs of OVT were found in a second follow-up CT scan in March 2022 (Figure 2). Ovarian vein diameter was 7 mm at that time. She currently continues on adjuvant TMX without any further events.

## Discussion

Reports on anticancer hormone therapy and/or targeted antineoplastic drugs as risk or associated factors for OVT are rare. A published large series on isolated gonadal vein thrombosis in oncology patients from one comprehensive cancer center in Texas included 21 out of 196 patients (10.7%) on hormone therapy, but there is a lack of details on specific diagnoses and drugs in those patients [4]. There is one published case report describing a BC patient who developed symptomatic right OVT during hormone therapy with anastrozole combined with targeted therapy with abemaciclib, a cyclin-dependent kinase 4/6 inhibitor [5].

A potential effect of TMX on the appearance of OVT may be related to ovarian stimulation by this drug. In fact, there is a published case of uterine venous plexus thrombosis in a patient on treatment with TMX [6], and this may be secondary to the well-known stimulatory effect of this drug on the uterus. Nevertheless, associations of OVT with hormone therapy using TMX are very uncommon. An exhaustive pharmacovigilance review of data from the Food and Drug Administration Adverse Event Reporting System (FAERS) analyzed 385 TMX-associated thromboembolic events, with no case of OVT [7]. We are unaware of any previous report indicating a potentially synergistic prothrombotic effect of TMX and TZB.

There is some ongoing debate on the appropriate therapy of incidental OVT in the oncology setting. A British guideline on this subject advised in 2012 against the systematic use of anticoagulation for OVT in cancer patients, except for cases with inferior vena cava extension or pulmonary embolism, but this advice is mainly oriented to OVT appearing after gynecologic cancer surgery [8]. Other authors made a similar recommendation, based on a large series of oncology patients, but with the advice to initiate anticoagulation in cases not related to previous surgery, as those are more likely to represent an atypical thrombosis and not simply a sequelae of surgery [9]. A retrospective cohort study performed a specific analysis on incidental gonadal vein thromboses diagnosed after CT imaging [10]. 57 out of 58 patients were females with OVT, most had cancer, and 72% were treated with anticoagulants after the diagnosis, with good results. A systematic review and meta-analysis of observational studies has been published on the safety and efficacy of anticoagulant treatment in patients with OVT [11]. Although in reference to the general population with OVT and not specifically for cancer patients, anticoagulation seems to be associated with nonsignificant trends toward better recanalization and lower rates of recurrent events. A scoping review on OVT has concluded that there are barriers precluding clear demarcations on management between cancer and noncancer patients with OVT, and also between asymptomatic and symptomatic cases [12]. The authors considered that OVT belongs to the spectrum of venous thromboembolism, both in puerperal and cancer settings, and that an ongoing International Registry on the Use of Direct Oral Anticoagulants for the Treatment of Unusual Site Thromboembolism (DUST) would determine the real value of direct oral anticoagulants in this context. Further advances on guidelines, expert opinions, consensus meetings, and data from trials and registries about detailed aspects of OVT, as well as on incidental and unusual sites of thrombosis, are awaited and will improve the care of patients with this complication.

In view of these considerations, anticoagulation might be regarded as an option in asymptomatic incidental OVT detected by CT scans in cancer patients. Therapy choice may depend on patient factors like previous VTD history and thrombophilia, bleeding risks, and institutional practice.

## Conclusions

OVT is an unusual site of VTD, but may be detected as an incidental image finding in asymptomatic BC patients on treatment with TMX and trastuzumab. Evolution was favorable without anticoagulant therapy in our case report, with thrombus disappearance and absence of recurrent thromboembolic events. However, a focused review of published literature on this subject indicates that anticoagulation is an adequate option to be considered for these patients.
